# 

**Published:** 1907-03-16

**Authors:** 


### EXAMINATIONS

### *Questions and Answers.*

**Question.**—How can I become a Midwife?

**Answer.**—"HOW TO BECOME A CERTIFIED MIDWIFE," by Dr. E. L. Appel (price 1/2 post free), fully explains the legal requirements of the Midwives Act, and is a most valuable guide to any Nurse desirous of taking up Midwifery.

**Question.**—How can I obtain an easy introduction to the theory and the various technical terms used in Midwifery?

**Answer.**—"SIMPLE INTRODUCTORY LESSONS IN MIDWIFERY," by M. Loane (price 1/2 post free), is designed for the use of those women who are unable to profit immediately by any ordinary course of Lectures on the subject.

**Question.**—Where can I obtain a complete knowledge of Midwifery?

**Answer.**—"A COMPLETE HANDBOOK OF MIDWIFERY," by Dr. J. K. Watson (price 6/4 post free), is the most complete and up-to-date book on the subject, and was especially written to meet the requirements of candidates entering for the C.M.B. Examination. It is very fully illustrated with excellent diagrams, and its use as a text-book should do much to ensure the success of any Nurse sitting for these Examinations.

**Question.**—I am wanting a Reference Book on Midwifery. I am a fully qualified Midwife.

**Answer.**—"A PRACTICAL HANDBOOK OF MIDWIFERY," by Francis Haultain, M.D. (price 6/3 post free), is a most valuable handbook for the practising Midwife, and should find a place in every Midwife's bag for immediate reference in cases of emergency.

**The Scientific Press, Ltd.,**

*Catalogue of Nursing Manuals sent post free on application.*

**Publishers of Nursing Text-Books, Charts, and Case-Books of all Descriptions.**

**28 & 29 SOUTHAMPTON STREET, STRAND, LONDON, W.C.**

